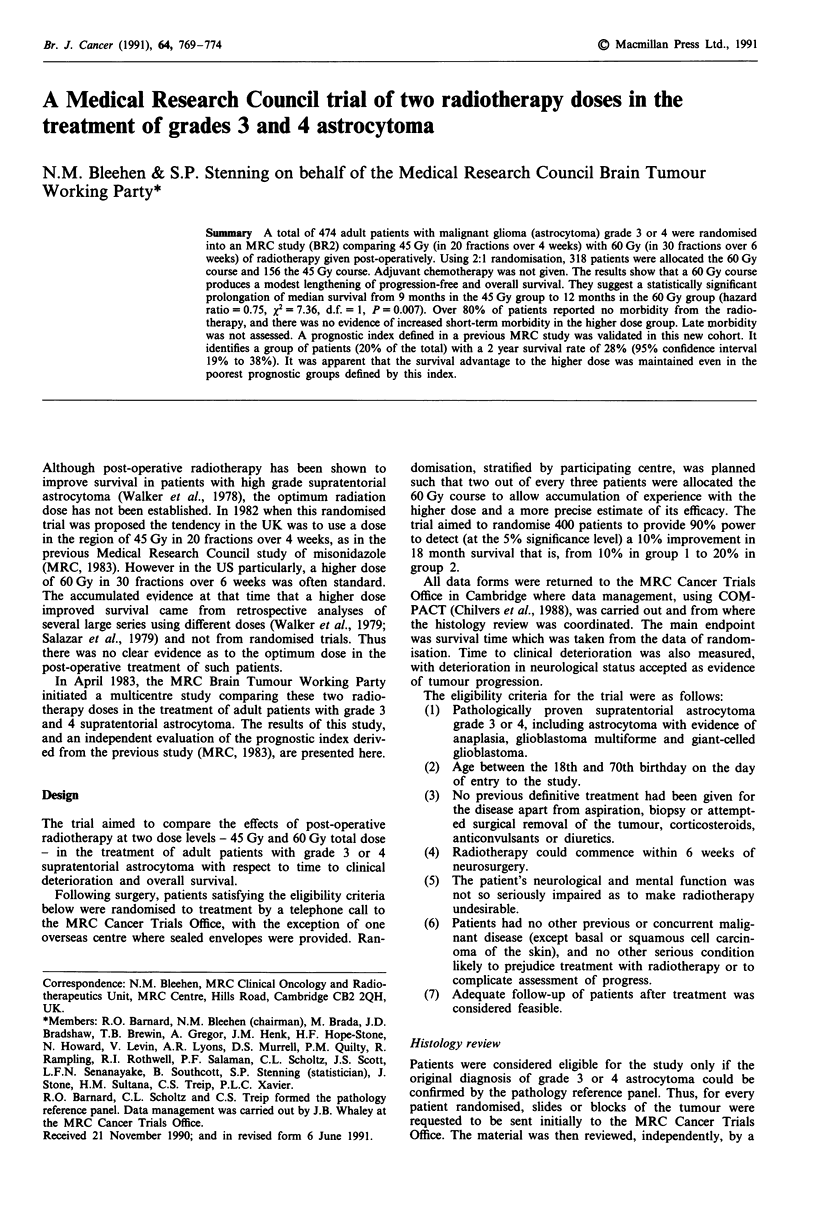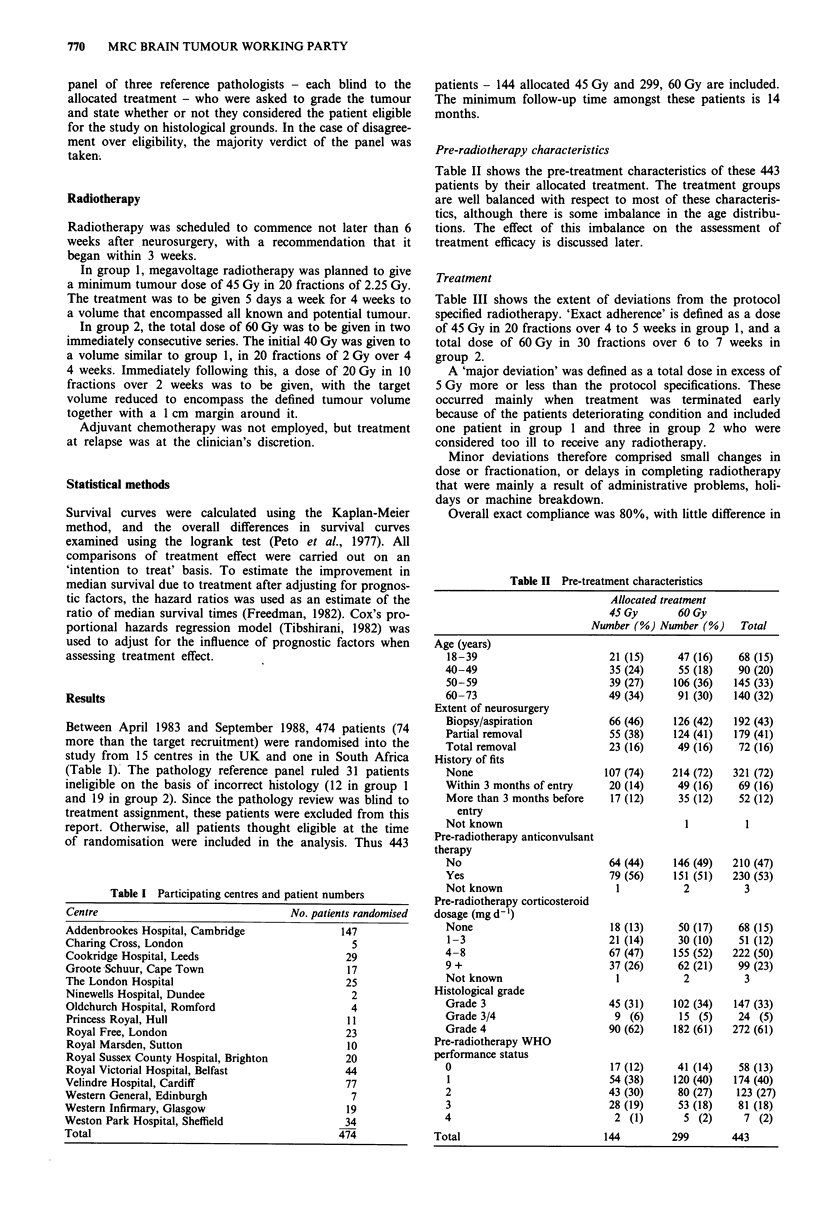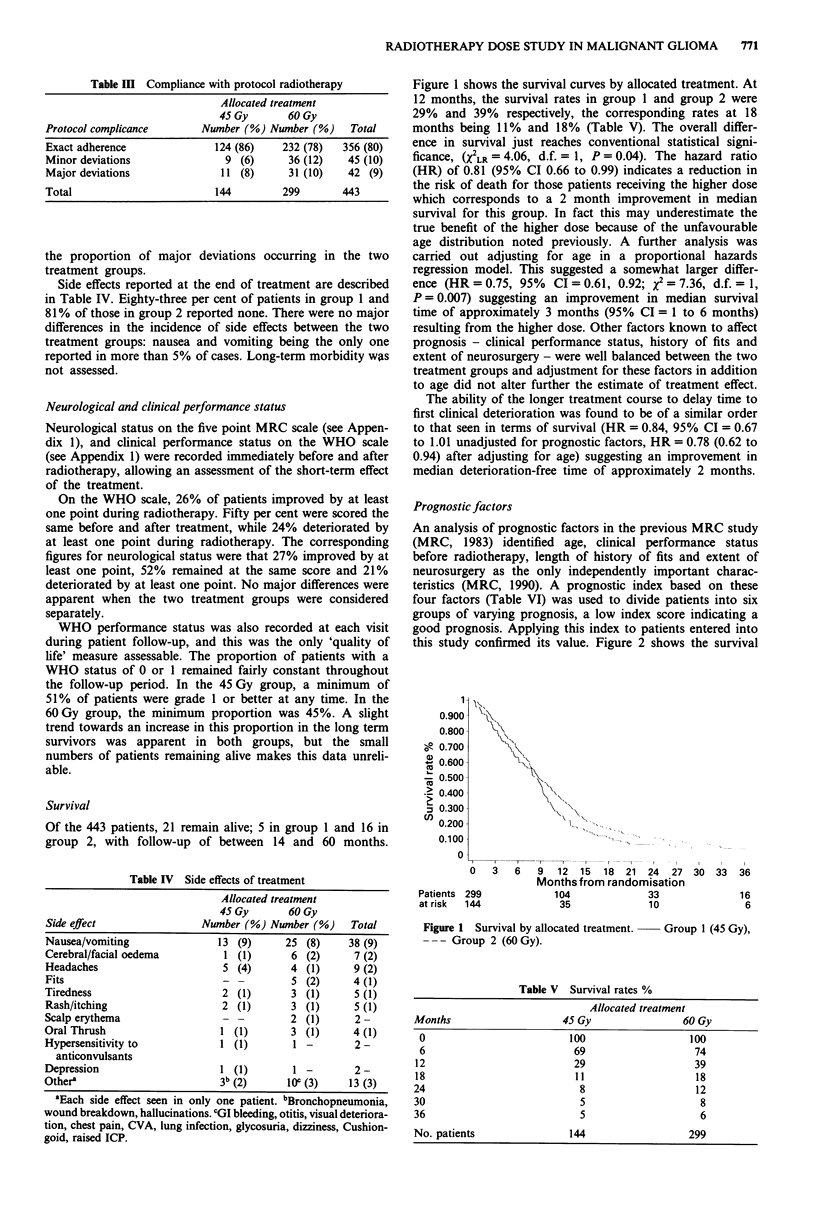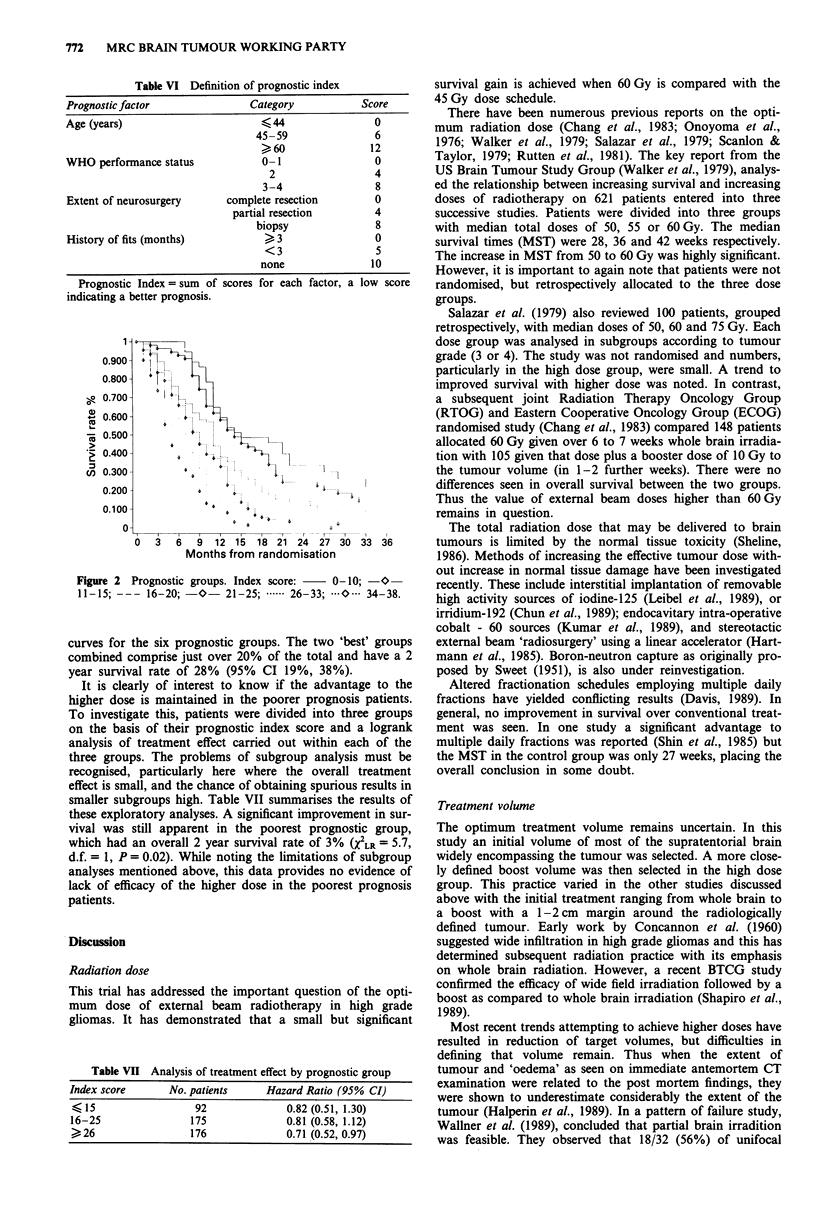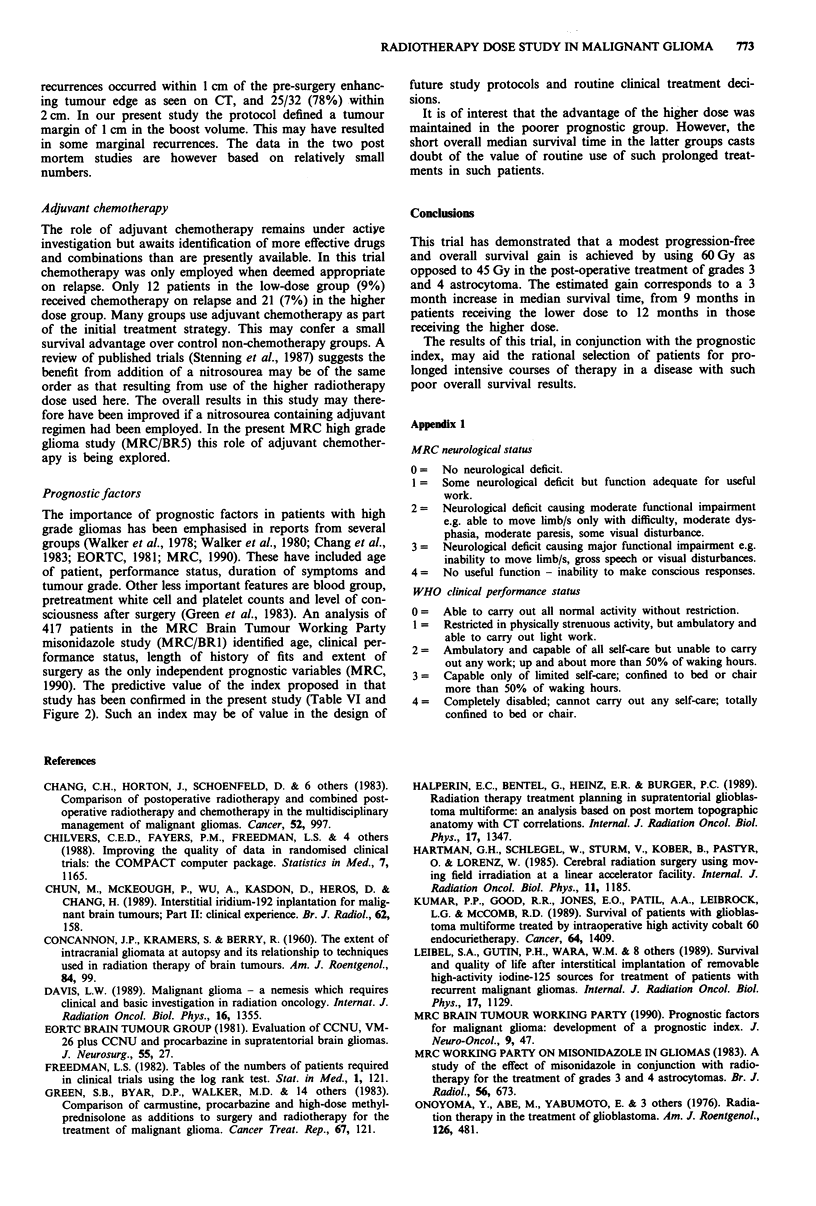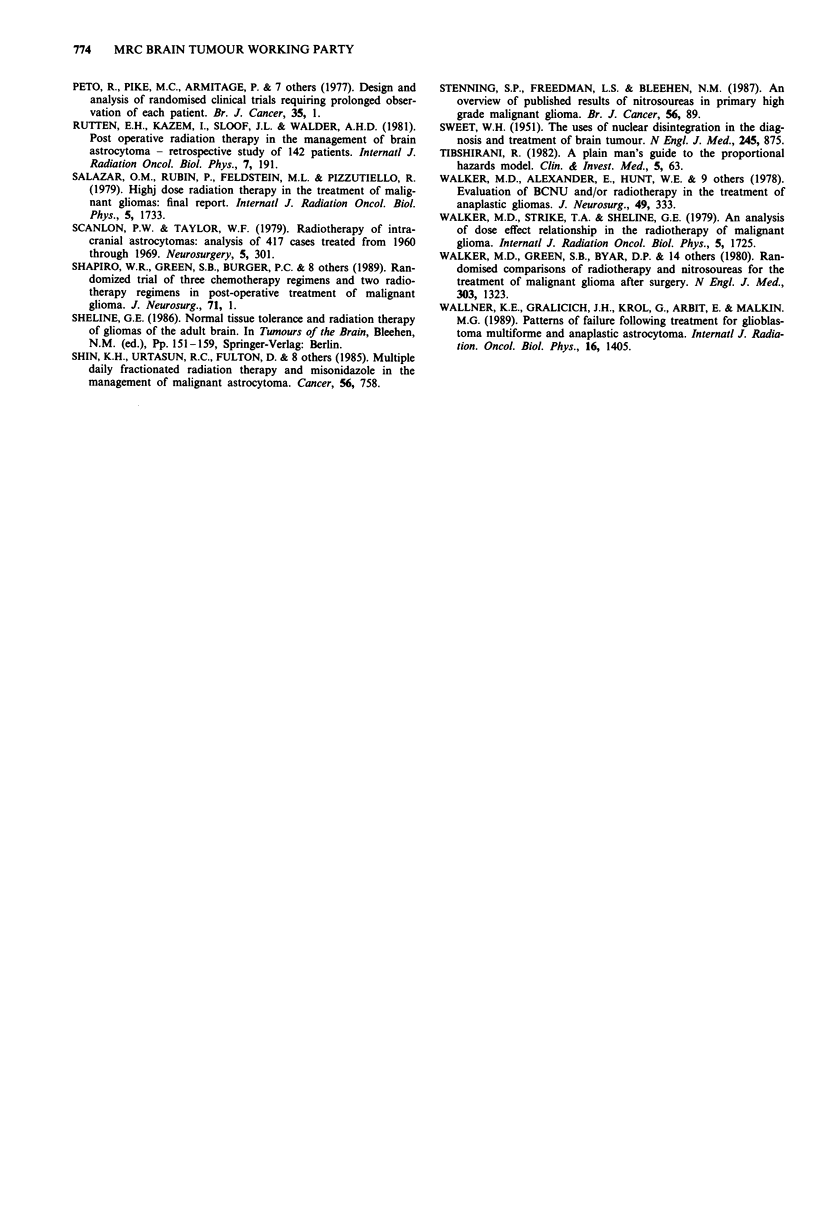# A Medical Research Council trial of two radiotherapy doses in the treatment of grades 3 and 4 astrocytoma. The Medical Research Council Brain Tumour Working Party.

**DOI:** 10.1038/bjc.1991.396

**Published:** 1991-10

**Authors:** N. M. Bleehen, S. P. Stenning

**Affiliations:** MRC Clinical Oncology and Radiotherapeutics Unit, MRC Centre, Cambridge, UK.

## Abstract

A total of 474 adult patients with malignant glioma (astrocytoma) grade 3 or 4 were randomised into an MRC study (BR2) comparing 45 Gy (in 20 fractions over 4 weeks) with 60 Gy (in 30 fractions over 6 weeks) of radiotherapy given post-operatively. Using 2:1 randomisation, 318 patients were allocated the 60 Gy course and 156 the 45 Gy course. Adjuvant chemotherapy was not given. The results show that a 60 Gy course produces a modest lengthening of progression-free and overall survival. They suggest a statistically significant prolongation of median survival from 9 months in the 45 Gy group to 12 months in the 60 Gy group (hazard ratio = 0.75, chi 2 = 7.36, d.f. = 1, P = 0.007). Over 80% of patients reported no morbidity from the radiotherapy, and there was no evidence of increased short-term morbidity in the higher dose group. Late morbidity was not assessed. A prognostic index defined in a previous MRC study was validated in this new cohort. It identifies a group of patients (20% of the total) with a 2 year survival rate of 28% (95% confidence interval 19% to 38%). It was apparent that the survival advantage to the higher dose was maintained even in the poorest prognostic groups defined by this index.


Br. J. Cancer (1991), 64, 769-774 © Macmillan Press Ltd., 1991

### A Medical Research Council trial of two radiotherapy doses in the treatment of grades 3 and 4 astrocytoma

N.M. Bleehen & S.P. Stenning on behalf of the Medical Research Council Brain Tumour Working Party*

**Summary** A total of 474 adult patients with malignant glioma (astrocytoma) grade 3 or 4 were randomised into an MRC study (BR2) comparing 45 Gy (in 20 fractions over 4 weeks) with 60 Gy (in 30 fractions over 6 weeks) of radiotherapy given post-operatively. Using 2:1 randomisation, 318 patients were allocated the 60 Gy course and 156 the 45 Gy course. Adjuvant chemotherapy was not given. The results show that a 60 Gy course produces a modest lengthening of progression-free and overall survival. They suggest a statistically significant prolongation of median survival from 9 months in the 45 Gy group to 12 months in the 60 Gy group (hazard ratio = 0.75, $\chi^2 = 7.36$, d.f. = 1, $P = 0.007$). Over 80% of patients reported no morbidity from the radiotherapy, and there was no evidence of increased short-term morbidity in the higher dose group. Late morbidity was not assessed. A prognostic index defined in a previous MRC study was validated in this new cohort. It identifies a group of patients (20% of the total) with a 2 year survival rate of 28% (95% confidence interval 19% to 38%). It was apparent that the survival advantage to the higher dose was maintained even in the poorest prognostic groups defined by this index.

Although post-operative radiotherapy has been shown to improve survival in patients with high grade supratentorial astrocytoma (Walker *et al.*, 1978), the optimum radiation dose has not been established. In 1982 when this randomised trial was proposed the tendency in the UK was to use a dose in the region of 45 Gy in 20 fractions over 4 weeks, as in the previous Medical Research Council study of misonidazole (MRC, 1983). However in the US particularly, a higher dose of 60 Gy in 30 fractions over 6 weeks was often standard. The accumulated evidence at that time that a higher dose improved survival came from retrospective analyses of several large series using different doses (Walker *et al.*, 1979; Salazar *et al.*, 1979) and not from randomised trials. Thus there was no clear evidence as to the optimum dose in the post-operative treatment of such patients.

In April 1983, the MRC Brain Tumour Working Party initiated a multicentre study comparing these two radiotherapy doses in the treatment of adult patients with grade 3 and 4 supratentorial astrocytoma. The results of this study, and an independent evaluation of the prognostic index derived from the previous study (MRC, 1983), are presented here.

### Design

The trial aimed to compare the effects of post-operative radiotherapy at two dose levels – 45 Gy and 60 Gy total dose – in the treatment of adult patients with grade 3 or 4 supratentorial astrocytoma with respect to time to clinical deterioration and overall survival.

Following surgery, patients satisfying the eligibility criteria below were randomised to treatment by a telephone call to the MRC Cancer Trials Office, with the exception of one overseas centre where sealed envelopes were provided. Randomisation, stratified by participating centre, was planned such that two out of every three patients were allocated the 60 Gy course to allow accumulation of experience with the higher dose and a more precise estimate of its efficacy. The trial aimed to randomise 400 patients to provide 90% power to detect (at the 5% significance level) a 10% improvement in 18 month survival that is, from 10% in group 1 to 20% in group 2.

All data forms were returned to the MRC Cancer Trials Office in Cambridge where data management, using COMPACT (Chilvers *et al.*, 1988), was carried out and from where the histology review was coordinated. The main endpoint was survival time which was taken from the data of randomisation. Time to clinical deterioration was also measured, with deterioration in neurological status accepted as evidence of tumour progression.

The eligibility criteria for the trial were as follows:

(1) Pathologically proven supratentorial astrocytoma grade 3 or 4, including astrocytoma with evidence of anaplasia, glioblastoma multiforme and giant-celled glioblastoma.
(2) Age between the 18th and 70th birthday on the day of entry to the study.
(3) No previous definitive treatment had been given for the disease apart from aspiration, biopsy or attempted surgical removal of the tumour, corticosteroids, anticonvulsants or diuretics.
(4) Radiotherapy could commence within 6 weeks of neurosurgery.
(5) The patient's neurological and mental function was not so seriously impaired as to make radiotherapy undesirable.
(6) Patients had no other previous or concurrent malignant disease (except basal or squamous cell carcinoma of the skin), and no other serious condition likely to prejudice treatment with radiotherapy or to complicate assessment of progress.
(7) Adequate follow-up of patients after treatment was considered feasible.

### *Histology review*

Patients were considered eligible for the study only if the original diagnosis of grade 3 or 4 astrocytoma could be confirmed by the pathology reference panel. Thus, for every patient randomised, slides or blocks of the tumour were requested to be sent initially to the MRC Cancer Trials Office. The material was then reviewed, independently, by a

Correspondence: N.M. Bleehen, MRC Clinical Oncology and Radiotherapeutics Unit, MRC Centre, Hills Road, Cambridge CB2 2QH, UK.

*Members: R.O. Barnard, N.M. Bleehen (chairman), M. Brada, J.D. Bradshaw, T.B. Brewin, A. Gregor, J.M. Henk, H.F. Hope-Stone, N. Howard, V. Levin, A.R. Lyons, D.S. Murrell, P.M. Quilty, R. Rampling, R.I. Rothwell, P.F. Salaman, C.L. Scholtz, J.S. Scott, L.F.N. Senanayake, B. Southcott, S.P. Stenning (statistician), J. Stone, H.M. Sultana, C.S. Treip, P.L.C. Xavier.

R.O. Barnard, C.L. Scholtz and C.S. Treip formed the pathology reference panel. Data management was carried out by J.B. Whaley at the MRC Cancer Trials Office.

Received 21 November 1990; and in revised form 6 June 1991.

panel of three reference pathologists – each blind to the allocated treatment – who were asked to grade the tumour and state whether or not they considered the patient eligible for the study on histological grounds. In the case of disagreement over eligibility, the majority verdict of the panel was taken.

### Radiotherapy

Radiotherapy was scheduled to commence not later than 6 weeks after neurosurgery, with a recommendation that it began within 3 weeks.

In group 1, megavoltage radiotherapy was planned to give a minimum tumour dose of 45 Gy in 20 fractions of 2.25 Gy. The treatment was to be given 5 days a week for 4 weeks to a volume that encompassed all known and potential tumour.

In group 2, the total dose of 60 Gy was to be given in two immediately consecutive series. The initial 40 Gy was given to a volume similar to group 1, in 20 fractions of 2 Gy over 4 4 weeks. Immediately following this, a dose of 20 Gy in 10 fractions over 2 weeks was to be given, with the target volume reduced to encompass the defined tumour volume together with a 1 cm margin around it.

Adjuvant chemotherapy was not employed, but treatment at relapse was at the clinician's discretion.

### Statistical methods

Survival curves were calculated using the Kaplan-Meier method, and the overall differences in survival curves examined using the logrank test (Peto *et al.*, 1977). All comparisons of treatment effect were carried out on an 'intention to treat' basis. To estimate the improvement in median survival due to treatment after adjusting for prognostic factors, the hazard ratios was used as an estimate of the ratio of median survival times (Freedman, 1982). Cox's proportional hazards regression model (Tibshirani, 1982) was used to adjust for the influence of prognostic factors when assessing treatment effect.

### Results

Between April 1983 and September 1988, 474 patients (74 more than the target recruitment) were randomised into the study from 15 centres in the UK and one in South Africa (Table I). The pathology reference panel ruled 31 patients ineligible on the basis of incorrect histology (12 in group 1 and 19 in group 2). Since the pathology review was blind to treatment assignment, these patients were excluded from this report. Otherwise, all patients thought eligible at the time of randomisation were included in the analysis. Thus 443 patients – 144 allocated 45 Gy and 299, 60 Gy are included. The minimum follow-up time amongst these patients is 14 months.

### *Pre-radiotherapy characteristics*

Table II shows the pre-treatment characteristics of these 443 patients by their allocated treatment. The treatment groups are well balanced with respect to most of these characteristics, although there is some imbalance in the age distributions. The effect of this imbalance on the assessment of treatment efficacy is discussed later.

### *Treatment*

Table III shows the extent of deviations from the protocol specified radiotherapy. 'Exact adherence' is defined as a dose of 45 Gy in 20 fractions over 4 to 5 weeks in group 1, and a total dose of 60 Gy in 30 fractions over 6 to 7 weeks in group 2.

A 'major deviation' was defined as a total dose in excess of 5 Gy more or less than the protocol specifications. These occurred mainly when treatment was terminated early because of the patients deteriorating condition and included one patient in group 1 and three in group 2 who were considered too ill to receive any radiotherapy.

Minor deviations therefore comprised small changes in dose or fractionation, or delays in completing radiotherapy that were mainly a result of administrative problems, holidays or machine breakdown.

Overall exact compliance was 80%, with little difference in

**Table I** Participating centres and patient numbers

| *Centre* | *No. patients randomised* |
|---|---|
| Addenbrookes Hospital, Cambridge | 147 |
| Charing Cross, London | 5 |
| Cookridge Hospital, Leeds | 29 |
| Groote Schuur, Cape Town | 17 |
| The London Hospital | 25 |
| Ninewells Hospital, Dundee | 2 |
| Oldchurch Hospital, Romford | 4 |
| Princess Royal, Hull | 11 |
| Royal Free, London | 23 |
| Royal Marsden, Sutton | 10 |
| Royal Sussex County Hospital, Brighton | 20 |
| Royal Victorial Hospital, Belfast | 44 |
| Velindre Hospital, Cardiff | 77 |
| Western General, Edinburgh | 7 |
| Western Infirmary, Glasgow | 19 |
| Weston Park Hospital, Sheffield | 34 |
| Total | 474 |

**Table II** Pre-treatment characteristics

| | *Allocated treatment* | | |
|---|---|---|---|
| | *45 Gy* | *60 Gy* | |
| | *Number (%)* | *Number (%)* | *Total* |
| Age (years) | | | |
| 18–39 | 21 (15) | 47 (16) | 68 (15) |
| 40–49 | 35 (24) | 55 (18) | 90 (20) |
| 50–59 | 39 (27) | 106 (36) | 145 (33) |
| 60–73 | 49 (34) | 91 (30) | 140 (32) |
| Extent of neurosurgery | | | |
| Biopsy/aspiration | 66 (46) | 126 (42) | 192 (43) |
| Partial removal | 55 (38) | 124 (41) | 179 (41) |
| Total removal | 23 (16) | 49 (16) | 72 (16) |
| History of fits | | | |
| None | 107 (74) | 214 (72) | 321 (72) |
| Within 3 months of entry | 20 (14) | 49 (16) | 69 (16) |
| More than 3 months before entry | 17 (12) | 35 (12) | 52 (12) |
| Not known | | 1 | 1 |
| Pre-radiotherapy anticonvulsant therapy | | | |
| No | 64 (44) | 146 (49) | 210 (47) |
| Yes | 79 (56) | 151 (51) | 230 (53) |
| Not known | 1 | 2 | 3 |
| Pre-radiotherapy corticosteroid dosage (mg $d^{-1}$) | | | |
| None | 18 (13) | 50 (17) | 68 (15) |
| 1–3 | 21 (14) | 30 (10) | 51 (12) |
| 4–8 | 67 (47) | 155 (52) | 222 (50) |
| 9+ | 37 (26) | 62 (21) | 99 (23) |
| Not known | 1 | 2 | 3 |
| Histological grade | | | |
| Grade 3 | 45 (31) | 102 (34) | 147 (33) |
| Grade 3/4 | 9 (6) | 15 (5) | 24 (5) |
| Grade 4 | 90 (62) | 182 (61) | 272 (61) |
| Pre-radiotherapy WHO performance status | | | |
| 0 | 17 (12) | 41 (14) | 58 (13) |
| 1 | 54 (38) | 120 (40) | 174 (40) |
| 2 | 43 (30) | 80 (27) | 123 (27) |
| 3 | 28 (19) | 53 (18) | 81 (18) |
| 4 | 2 (1) | 5 (2) | 7 (2) |
| Total | 144 | 299 | 443 |

**Table III** Compliance with protocol radiotherapy

| *Protocol complicance* | *Allocated treatment* 45 Gy Number (%) | 60 Gy Number (%) | *Total* |
|---|---|---|---|
| Exact adherence | 124 (86) | 232 (78) | 356 (80) |
| Minor deviations | 9 (6) | 36 (12) | 45 (10) |
| Major deviations | 11 (8) | 31 (10) | 42 (9) |
| Total | 144 | 299 | 443 |

the proportion of major deviations occurring in the two treatment groups.

Side effects reported at the end of treatment are described in Table IV. Eighty-three per cent of patients in group 1 and 81% of those in group 2 reported none. There were no major differences in the incidence of side effects between the two treatment groups: nausea and vomiting being the only one reported in more than 5% of cases. Long-term morbidity was not assessed.

### *Neurological and clinical performance status*

Neurological status on the five point MRC scale (see Appendix 1), and clinical performance status on the WHO scale (see Appendix 1) were recorded immediately before and after radiotherapy, allowing an assessment of the short-term effect of the treatment.

On the WHO scale, 26% of patients improved by at least one point during radiotherapy. Fifty per cent were scored the same before and after treatment, while 24% deteriorated by at least one point during radiotherapy. The corresponding figures for neurological status were that 27% improved by at least one point, 52% remained at the same score and 21% deteriorated by at least one point. No major differences were apparent when the two treatment groups were considered separately.

WHO performance status was also recorded at each visit during patient follow-up, and this was the only 'quality of life' measure assessable. The proportion of patients with a WHO status of 0 or 1 remained fairly constant throughout the follow-up period. In the 45 Gy group, a minimum of 51% of patients were grade 1 or better at any time. In the 60 Gy group, the minimum proportion was 45%. A slight trend towards an increase in this proportion in the long term survivors was apparent in both groups, but the small numbers of patients remaining alive makes this data unreliable.

### *Survival*

Of the 443 patients, 21 remain alive; 5 in group 1 and 16 in group 2, with follow-up of between 14 and 60 months.

**Table IV** Side effects of treatment

| *Side effect* | *Allocated treatment* 45 Gy Number (%) | 60 Gy Number (%) | *Total* |
|---|---|---|---|
| Nausea/vomiting | 13 (9) | 25 (8) | 38 (9) |
| Cerebral/facial oedema | 1 (1) | 6 (2) | 7 (2) |
| Headaches | 5 (4) | 4 (1) | 9 (2) |
| Fits | – – | 5 (2) | 4 (1) |
| Tiredness | 2 (1) | 3 (1) | 5 (1) |
| Rash/itching | 2 (1) | 3 (1) | 5 (1) |
| Scalp erythema | – – | 2 (1) | 2 – |
| Oral Thrush | 1 (1) | 3 (1) | 4 (1) |
| Hypersensitivity to anticonvulsants | 1 (1) | 1 – | 2 – |
| Depression | 1 (1) | 1 – | 2 – |
| Other[a] | 3[b] (2) | 10[c] (3) | 13 (3) |

[a]Each side effect seen in only one patient. [b]Bronchopneumonia, wound breakdown, hallucinations. [c]GI bleeding, otitis, visual deterioration, chest pain, CVA, lung infection, glycosuria, dizziness, Cushiongoid, raised ICP.

Figure 1 shows the survival curves by allocated treatment. At 12 months, the survival rates in group 1 and group 2 were 29% and 39% respectively, the corresponding rates at 18 months being 11% and 18% (Table V). The overall difference in survival just reaches conventional statistical significance, ($\chi^2_{LR} = 4.06$, d.f. = 1, $P = 0.04$). The hazard ratio (HR) of 0.81 (95% CI 0.66 to 0.99) indicates a reduction in the risk of death for those patients receiving the higher dose which corresponds to a 2 month improvement in median survival for this group. In fact this may underestimate the true benefit of the higher dose because of the unfavourable age distribution noted previously. A further analysis was carried out adjusting for age in a proportional hazards regression model. This suggested a somewhat larger difference (HR = 0.75, 95% CI = 0.61, 0.92; $\chi^2 = 7.36$, d.f. = 1, $P = 0.007$) suggesting an improvement in median survival time of approximately 3 months (95% CI = 1 to 6 months) resulting from the higher dose. Other factors known to affect prognosis – clinical performance status, history of fits and extent of neurosurgery – were well balanced between the two treatment groups and adjustment for these factors in addition to age did not alter further the estimate of treatment effect.

The ability of the longer treatment course to delay time to first clinical deterioration was found to be of a similar order to that seen in terms of survival (HR = 0.84, 95% CI = 0.67 to 1.01 unadjusted for prognostic factors, HR = 0.78 (0.62 to 0.94) after adjusting for age) suggesting an improvement in median deterioration-free time of approximately 2 months.

### *Prognostic factors*

An analysis of prognostic factors in the previous MRC study (MRC, 1983) identified age, clinical performance status before radiotherapy, length of history of fits and extent of neurosurgery as the only independently important characteristics (MRC, 1990). A prognostic index based on these four factors (Table VI) was used to divide patients into six groups of varying prognosis, a low index score indicating a good prognosis. Applying this index to patients entered into this study confirmed its value. Figure 2 shows the survival

**Figure 1** Survival by allocated treatment. —— Group 1 (45 Gy), --- Group 2 (60 Gy).

**Table V** Survival rates %

| *Months* | *Allocated treatment* 45 Gy | 60 Gy |
|---|---|---|
| 0 | 100 | 100 |
| 6 | 69 | 74 |
| 12 | 29 | 39 |
| 18 | 11 | 18 |
| 24 | 8 | 12 |
| 30 | 5 | 8 |
| 36 | 5 | 6 |
| No. patients | 144 | 299 |

**Table VI** Definition of prognostic index

| *Prognostic factor* | *Category* | *Score* |
|---|---|---|
| Age (years) | ⩽44 | 0 |
| | 45–59 | 6 |
| | ⩾60 | 12 |
| WHO performance status | 0–1 | 0 |
| | 2 | 4 |
| | 3–4 | 8 |
| Extent of neurosurgery | complete resection | 0 |
| | partial resection | 4 |
| | biopsy | 8 |
| History of fits (months) | ⩾3 | 0 |
| | <3 | 5 |
| | none | 10 |

Prognostic Index = sum of scores for each factor, a low score indicating a better prognosis.

**Figure 2** Prognostic groups. Index score: —— 0–10; —◇— 11–15; --- 16–20; —◇— 21–25; ······ 26–33; ···◇··· 34–38.

curves for the six prognostic groups. The two 'best' groups combined comprise just over 20% of the total and have a 2 year survival rate of 28% (95% CI 19%, 38%).

It is clearly of interest to know if the advantage to the higher dose is maintained in the poorer prognosis patients. To investigate this, patients were divided into three groups on the basis of their prognostic index score and a logrank analysis of treatment effect carried out within each of the three groups. The problems of subgroup analysis must be recognised, particularly here where the overall treatment effect is small, and the chance of obtaining spurious results in smaller subgroups high. Table VII summarises the results of these exploratory analyses. A significant improvement in survival was still apparent in the poorest prognostic group, which had an overall 2 year survival rate of 3% ($\chi^2_{LR} = 5.7$, d.f. = 1, $P = 0.02$). While noting the limitations of subgroup analyses mentioned above, this data provides no evidence of lack of efficacy of the higher dose in the poorest prognosis patients.

### Discussion

### *Radiation dose*

This trial has addressed the important question of the optimum dose of external beam radiotherapy in high grade gliomas. It has demonstrated that a small but significant survival gain is achieved when 60 Gy is compared with the 45 Gy dose schedule.

There have been numerous previous reports on the optimum radiation dose (Chang *et al.*, 1983; Onoyoma *et al.*, 1976; Walker *et al.*, 1979; Salazar *et al.*, 1979; Scanlon & Taylor, 1979; Rutten *et al.*, 1981). The key report from the US Brain Tumour Study Group (Walker *et al.*, 1979), analysed the relationship between increasing survival and increasing doses of radiotherapy on 621 patients entered into three successive studies. Patients were divided into three groups with median total doses of 50, 55 or 60 Gy. The median survival times (MST) were 28, 36 and 42 weeks respectively. The increase in MST from 50 to 60 Gy was highly significant. However, it is important to again note that patients were not randomised, but retrospectively allocated to the three dose groups.

Salazar *et al.* (1979) also reviewed 100 patients, grouped retrospectively, with median doses of 50, 60 and 75 Gy. Each dose group was analysed in subgroups according to tumour grade (3 or 4). The study was not randomised and numbers, particularly in the high dose group, were small. A trend to improved survival with higher dose was noted. In contrast, a subsequent joint Radiation Therapy Oncology Group (RTOG) and Eastern Cooperative Oncology Group (ECOG) randomised study (Chang *et al.*, 1983) compared 148 patients allocated 60 Gy given over 6 to 7 weeks whole brain irradiation with 105 given that dose plus a booster dose of 10 Gy to the tumour volume (in 1–2 further weeks). There were no differences seen in overall survival between the two groups. Thus the value of external beam doses higher than 60 Gy remains in question.

The total radiation dose that may be delivered to brain tumours is limited by the normal tissue toxicity (Sheline, 1986). Methods of increasing the effective tumour dose without increase in normal tissue damage have been investigated recently. These include interstitial implantation of removable high activity sources of iodine-125 (Leibel *et al.*, 1989), or irridium-192 (Chun *et al.*, 1989); endocavitary intra-operative cobalt - 60 sources (Kumar *et al.*, 1989), and stereotactic external beam 'radiosurgery' using a linear accelerator (Hartmann *et al.*, 1985). Boron-neutron capture as originally proposed by Sweet (1951), is also under reinvestigation.

Altered fractionation schedules employing multiple daily fractions have yielded conflicting results (Davis, 1989). In general, no improvement in survival over conventional treatment was seen. In one study a significant advantage to multiple daily fractions was reported (Shin *et al.*, 1985) but the MST in the control group was only 27 weeks, placing the overall conclusion in some doubt.

### *Treatment volume*

The optimum treatment volume remains uncertain. In this study an initial volume of most of the supratentorial brain widely encompassing the tumour was selected. A more closely defined boost volume was then selected in the high dose group. This practice varied in the other studies discussed above with the initial treatment ranging from whole brain to a boost with a 1–2 cm margin around the radiologically defined tumour. Early work by Concannon *et al.* (1960) suggested wide infiltration in high grade gliomas and this has determined subsequent radiation practice with its emphasis on whole brain radiation. However, a recent BTCG study confirmed the efficacy of wide field irradiation followed by a boost as compared to whole brain irradiation (Shapiro *et al.*, 1989).

Most recent trends attempting to achieve higher doses have resulted in reduction of target volumes, but difficulties in defining that volume remain. Thus when the extent of tumour and 'oedema' as seen on immediate antemortem CT examination were related to the post mortem findings, they were shown to underestimate considerably the extent of the tumour (Halperin *et al.*, 1989). In a pattern of failure study, Wallner *et al.* (1989), concluded that partial brain irradition was feasible. They observed that 18/32 (56%) of unifocal

**Table VII** Analysis of treatment effect by prognostic group

| *Index score* | *No. patients* | *Hazard Ratio (95% CI)* |
|---|---|---|
| ⩽15 | 92 | 0.82 (0.51, 1.30) |
| 16–25 | 175 | 0.81 (0.58, 1.12) |
| ⩾26 | 176 | 0.71 (0.52, 0.97) |

recurrences occurred within 1 cm of the pre-surgery enhancing tumour edge as seen on CT, and 25/32 (78%) within 2 cm. In our present study the protocol defined a tumour margin of 1 cm in the boost volume. This may have resulted in some marginal recurrences. The data in the two post mortem studies are however based on relatively small numbers.

### *Adjuvant chemotherapy*

The role of adjuvant chemotherapy remains under active investigation but awaits identification of more effective drugs and combinations than are presently available. In this trial chemotherapy was only employed when deemed appropriate on relapse. Only 12 patients in the low-dose group (9%) received chemotherapy on relapse and 21 (7%) in the higher dose group. Many groups use adjuvant chemotherapy as part of the initial treatment strategy. This may confer a small survival advantage over control non-chemotherapy groups. A review of published trials (Stenning *et al.*, 1987) suggests the benefit from addition of a nitrosourea may be of the same order as that resulting from use of the higher radiotherapy dose used here. The overall results in this study may therefore have been improved if a nitrosourea containing adjuvant regimen had been employed. In the present MRC high grade glioma study (MRC/BR5) this role of adjuvant chemotherapy is being explored.

### *Prognostic factors*

The importance of prognostic factors in patients with high grade gliomas has been emphasised in reports from several groups (Walker *et al.*, 1978; Walker *et al.*, 1980; Chang *et al.*, 1983; EORTC, 1981; MRC, 1990). These have included age of patient, performance status, duration of symptoms and tumour grade. Other less important features are blood group, pretreatment white cell and platelet counts and level of consciousness after surgery (Green *et al.*, 1983). An analysis of 417 patients in the MRC Brain Tumour Working Party misonidazole study (MRC/BR1) identified age, clinical performance status, length of history of fits and extent of surgery as the only independent prognostic variables (MRC, 1990). The predictive value of the index proposed in that study has been confirmed in the present study (Table VI and Figure 2). Such an index may be of value in the design of future study protocols and routine clinical treatment decisions.

It is of interest that the advantage of the higher dose was maintained in the poorer prognostic group. However, the short overall median survival time in the latter groups casts doubt of the value of routine use of such prolonged treatments in such patients.

### Conclusions

This trial has demonstrated that a modest progression-free and overall survival gain is achieved by using 60 Gy as opposed to 45 Gy in the post-operative treatment of grades 3 and 4 astrocytoma. The estimated gain corresponds to a 3 month increase in median survival time, from 9 months in patients receiving the lower dose to 12 months in those receiving the higher dose.

The results of this trial, in conjunction with the prognostic index, may aid the rational selection of patients for prolonged intensive courses of therapy in a disease with such poor overall survival results.

### Appendix 1

*MRC neurological status*

0 = No neurological deficit.
1 = Some neurological deficit but function adequate for useful work.
2 = Neurological deficit causing moderate functional impairment e.g. able to move limb/s only with difficulty, moderate dysphasia, moderate paresis, some visual disturbance.
3 = Neurological deficit causing major functional impairment e.g. inability to move limb/s, gross speech or visual disturbances.
4 = No useful function – inability to make conscious responses.

*WHO clinical performance status*

0 = Able to carry out all normal activity without restriction.
1 = Restricted in physically strenuous activity, but ambulatory and able to carry out light work.
2 = Ambulatory and capable of all self-care but unable to carry out any work; up and about more than 50% of waking hours.
3 = Capable only of limited self-care; confined to bed or chair more than 50% of waking hours.
4 = Completely disabled; cannot carry out any self-care; totally confined to bed or chair.

### References

CHANG, C.H., HORTON, J., SCHOENFELD, D. & 6 others (1983). Comparison of postoperative radiotherapy and combined postoperative radiotherapy and chemotherapy in the multidisciplinary management of malignant gliomas. *Cancer*, **52**, 997.

CHILVERS, C.E.D., FAYERS, P.M., FREEDMAN, L.S. & 4 others (1988). Improving the quality of data in randomised clinical trials: the COMPACT computer package. *Statistics in Med.*, **7**, 1165.

CHUN, M., MCKEOUGH, P., WU, A., KASDON, D., HEROS, D. & CHANG, H. (1989). Interstitial iridium-192 inplantation for malignant brain tumours; Part II: clinical experience. *Br. J. Radiol.*, **62**, 158.

CONCANNON, J.P., KRAMERS, S. & BERRY, R. (1960). The extent of intracranial gliomata at autopsy and its relationship to techniques used in radiation therapy of brain tumours. *Am. J. Roentgenol.*, **84**, 99.

DAVIS, L.W. (1989). Malignant glioma – a nemesis which requires clinical and basic investigation in radiation oncology. *Internat. J. Radiation Oncol. Biol. Phys.*, **16**, 1355.

EORTC BRAIN TUMOUR GROUP (1981). Evaluation of CCNU, VM-26 plus CCNU and procarbazine in supratentorial brain gliomas. *J. Neurosurg.*, **55**, 27.

FREEDMAN, L.S. (1982). Tables of the numbers of patients required in clinical trials using the log rank test. *Stat. in Med.*, **1**, 121.

GREEN, S.B., BYAR, D.P., WALKER, M.D. & 14 others (1983). Comparison of carmustine, procarbazine and high-dose methylprednisolone as additions to surgery and radiotherapy for the treatment of malignant glioma. *Cancer Treat. Rep.*, **67**, 121.

HALPERIN, E.C., BENTEL, G., HEINZ, E.R. & BURGER, P.C. (1989). Radiation therapy treatment planning in supratentorial glioblastoma multiforme: an analysis based on post mortem topographic anatomy with CT correlations. *Internat. J. Radiation Oncol. Biol. Phys.*, **17**, 1347.

HARTMAN, G.H., SCHLEGEL, W., STURM, V., KOBER, B., PASTYR, O. & LORENZ, W. (1985). Cerebral radiation surgery using moving field irradiation at a linear accelerator facility. *Internat. J. Radiation Oncol. Biol. Phys.*, **11**, 1185.

KUMAR, P.P., GOOD, R.R., JONES, E.O., PATIL, A.A., LEIBROCK, L.G. & MCCOMB, R.D. (1989). Survival of patients with glioblastoma multiforme treated by intraoperative high activity cobalt 60 endocurietherapy. *Cancer*, **64**, 1409.

LEIBEL, S.A., GUTIN, P.H., WARA, W.M. & 8 others (1989). Survival and quality of life after interstitical implantation of removable high-activity iodine-125 sources for treatment of patients with recurrent malignant gliomas. *Internat. J. Radiation Oncol. Biol. Phys.*, **17**, 1129.

MRC BRAIN TUMOUR WORKING PARTY (1990). Prognostic factors for malignant glioma: development of a prognostic index. *J. Neuro-Oncol.*, **9**, 47.

MRC WORKING PARTY ON MISONIDAZOLE IN GLIOMAS (1983). A study of the effect of misonidazole in conjunction with radiotherapy for the treatment of grades 3 and 4 astrocytomas. *Br. J. Radiol.*, **56**, 673.

ONOYOMA, Y., ABE, M., YABUMOTO, E. & 3 others (1976). Radiation therapy in the treatment of glioblastoma. *Am. J. Roentgenol.*, **126**, 481.

PETO, R., PIKE, M.C., ARMITAGE, P. & 7 others (1977). Design and analysis of randomised clinical trials requiring prolonged observation of each patient. *Br. J. Cancer*, **35**, 1.

RUTTEN, E.H., KAZEM, I., SLOOF, J.L. & WALDER, A.H.D. (1981). Post operative radiation therapy in the management of brain astrocytoma – retrospective study of 142 patients. *Internatl J. Radiation Oncol. Biol. Phys.*, **7**, 191.

SALAZAR, O.M., RUBIN, P., FELDSTEIN, M.L. & PIZZUTIELLO, R. (1979). Highj dose radiation therapy in the treatment of malignant gliomas: final report. *Internatl J. Radiation Oncol. Biol. Phys.*, **5**, 1733.

SCANLON, P.W. & TAYLOR, W.F. (1979). Radiotherapy of intracranial astrocytomas: analysis of 417 cases treated from 1960 through 1969. *Neurosurgery*, **5**, 301.

SHAPIRO, W.R., GREEN, S.B., BURGER, P.C. & 8 others (1989). Randomized trial of three chemotherapy regimens and two radiotherapy regimens in post-operative treatment of malignant glioma. *J. Neurosurg.*, **71**, 1.

SHELINE, G.E. (1986). Normal tissue tolerance and radiation therapy of gliomas of the adult brain. In *Tumours of the Brain*, Bleehen, N.M. (ed.), Pp. 151–159, Springer-Verlag: Berlin.

SHIN, K.H., URTASUN, R.C., FULTON, D. & 8 others (1985). Multiple daily fractionated radiation therapy and misonidazole in the management of malignant astrocytoma. *Cancer*, **56**, 758.

STENNING, S.P., FREEDMAN, L.S. & BLEEHEN, N.M. (1987). An overview of published results of nitrosoureas in primary high grade malignant glioma. *Br. J. Cancer*, **56**, 89.

SWEET, W.H. (1951). The uses of nuclear disintegration in the diagnosis and treatment of brain tumour. *N Engl. J. Med.*, **245**, 875.

TIBSHIRANI, R. (1982). A plain man's guide to the proportional hazards model. *Clin. & Invest. Med.*, **5**, 63.

WALKER, M.D., ALEXANDER, E., HUNT, W.E. & 9 others (1978). Evaluation of BCNU and/or radiotherapy in the treatment of anaplastic gliomas. *J. Neurosurg.*, **49**, 333.

WALKER, M.D., STRIKE, T.A. & SHELINE, G.E. (1979). An analysis of dose effect relationship in the radiotherapy of malignant glioma. *Internatl J. Radiation Oncol. Biol. Phys.*, **5**, 1725.

WALKER, M.D., GREEN, S.B., BYAR, D.P. & 14 others (1980). Randomised comparisons of radiotherapy and nitrosoureas for the treatment of malignant glioma after surgery. *N Engl. J. Med.*, **303**, 1323.

WALLNER, K.E., GRALICICH, J.H., KROL, G., ARBIT, E. & MALKIN. M.G. (1989). Patterns of failure following treatment for glioblastoma multiforme and anaplastic astrocytoma. *Internatl J. Radiation. Oncol. Biol. Phys.*, **16**, 1405.